# Supplementary figures and images for: The latency-associated transcript locus of herpes simplex virus 1 is a virulence determinant in human skin
Source: PLoS Pathog. 2020 Dec 28;16(12):e1009166. doi: 10.1371/journal.ppat.1009166 (PMC7794027; doi:10.1371/journal.ppat.1009166)

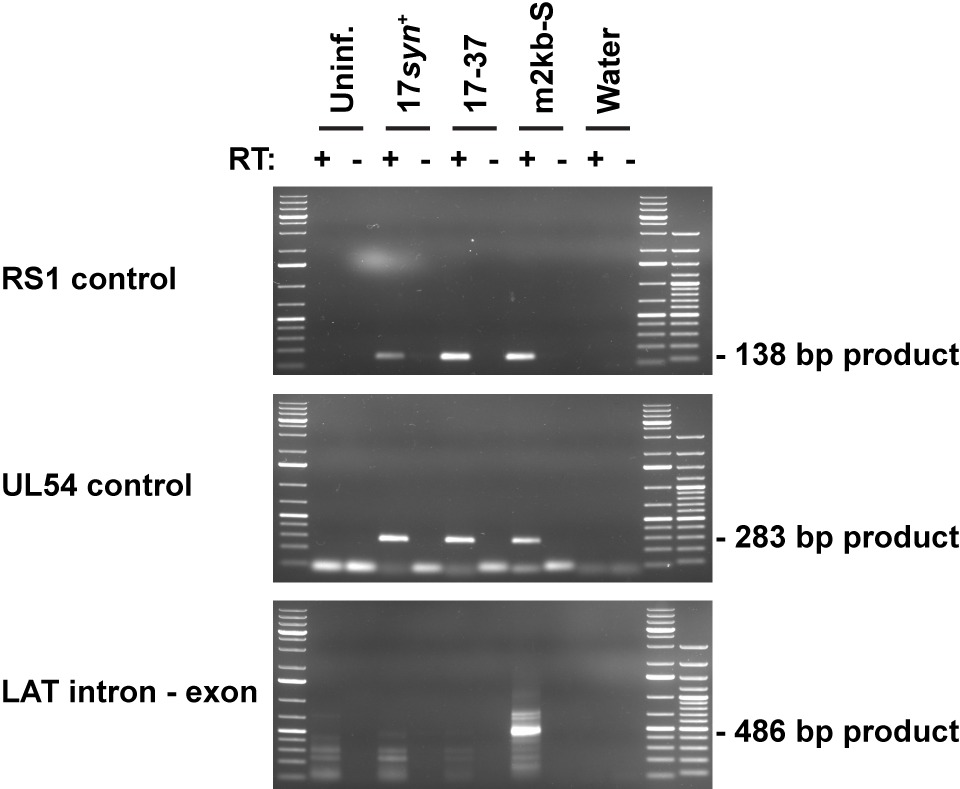

Supplement: S1 Fig — Splicing of 2 kb LAT intron during 17syn+, 17-37, and m2kb-S infection in Vero cells at 24 HPI (MOI 1). RT-PCR using gene-specific primers with or without reverse transcriptase (RT) was performed to verify lack of 2 kb LAT intron splicing in the splice mutant virus m2kb-S. Uninfected cells and water only were used as negative controls for all viral transcripts. RS1 and UL54 RNAs were detected as positive controls for viral infection. (TIF) [file ppat.1009166.s006.tif]

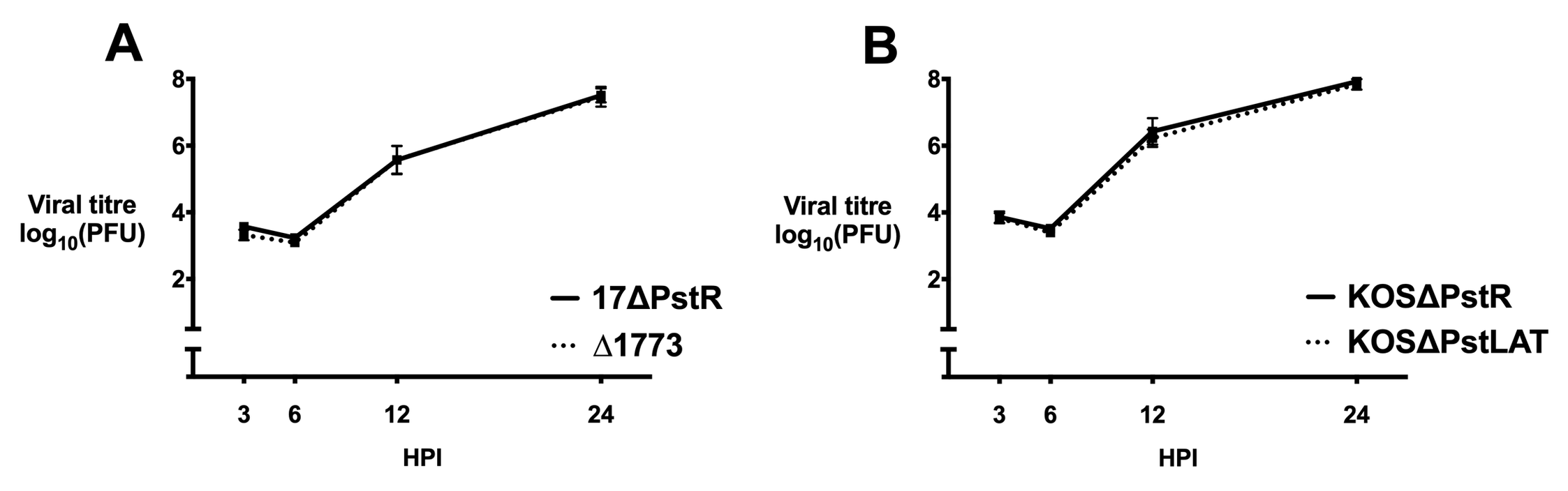

Supplement: S2 Fig — Viral titres (log10 PFU/ml ± SEM) from replication assays in HFs at specified time points 3, 6, 12, and 24 HPI (MOI 0.5; n = 3 for both viruses per time point). 2-way ANOVA was performed with Sidak’s correction, where all time points had non-significant P > 0.05 differences. (A) Δ1773 and 17ΔPstR and (B) KOSΔPstLAT and rescue KOSΔPstR virus comparison. (TIF) [file ppat.1009166.s007.tif]

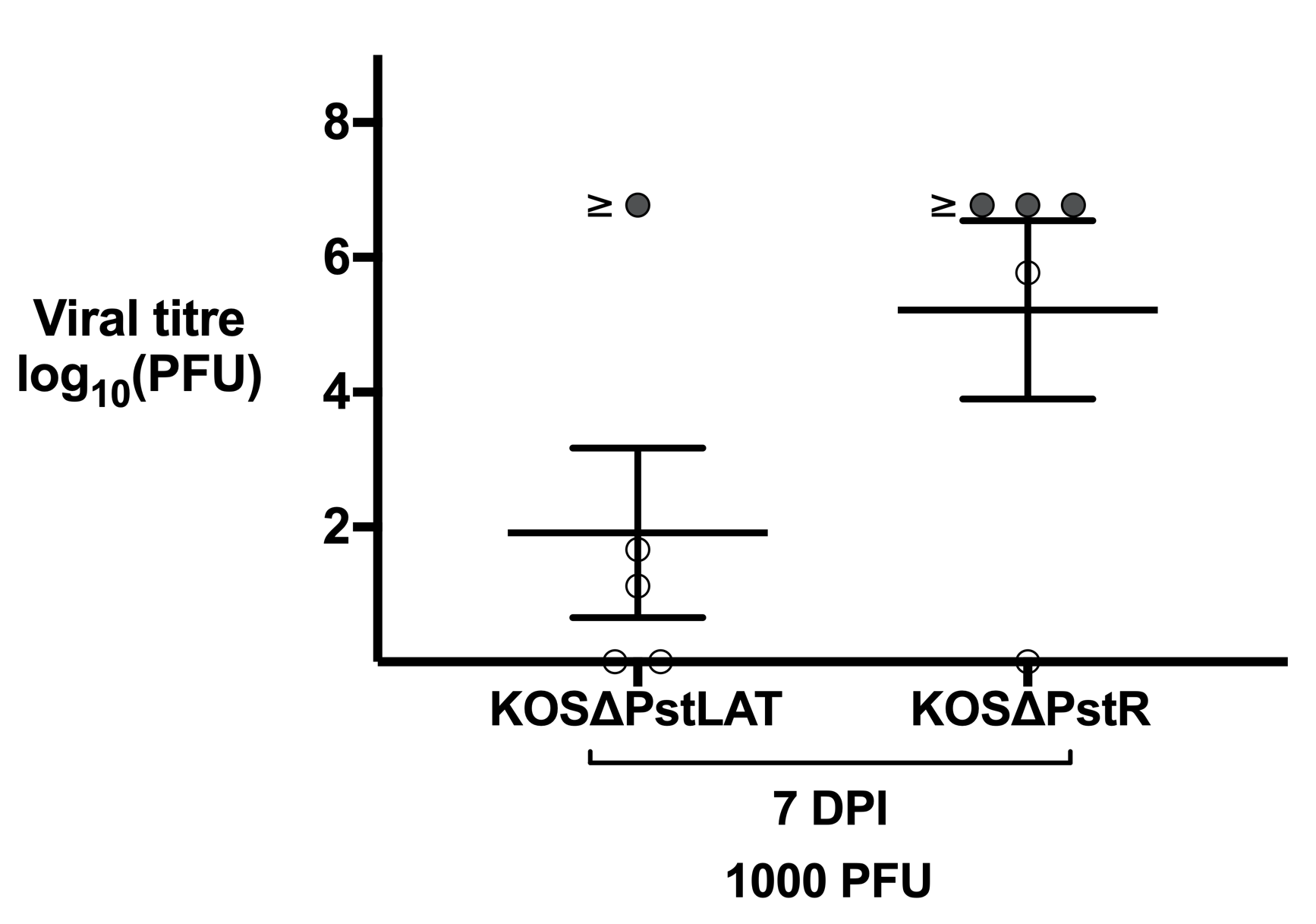

Supplement: S3 Fig — Viral titres (log10 PFU/implant ± SEM; each data point represents an individual replicate) recovered from in vivo skin xenografts infected with 1,000 PFU KOSΔPstLAT and KOSΔPstR at 7 DPI (n = 5). In this assay, we could not capture the exact values for four datapoints (one of KOSΔPstLAT and three of KOSΔPstR) because their titres were higher than the set detection ability in the particular experiment. Therefore, the datapoints in the figure are set at the maximum value that could have been possible to detect (circles filled with grey colour). No statistical analysis was done without the exact values. (TIF) [file ppat.1009166.s008.tif]

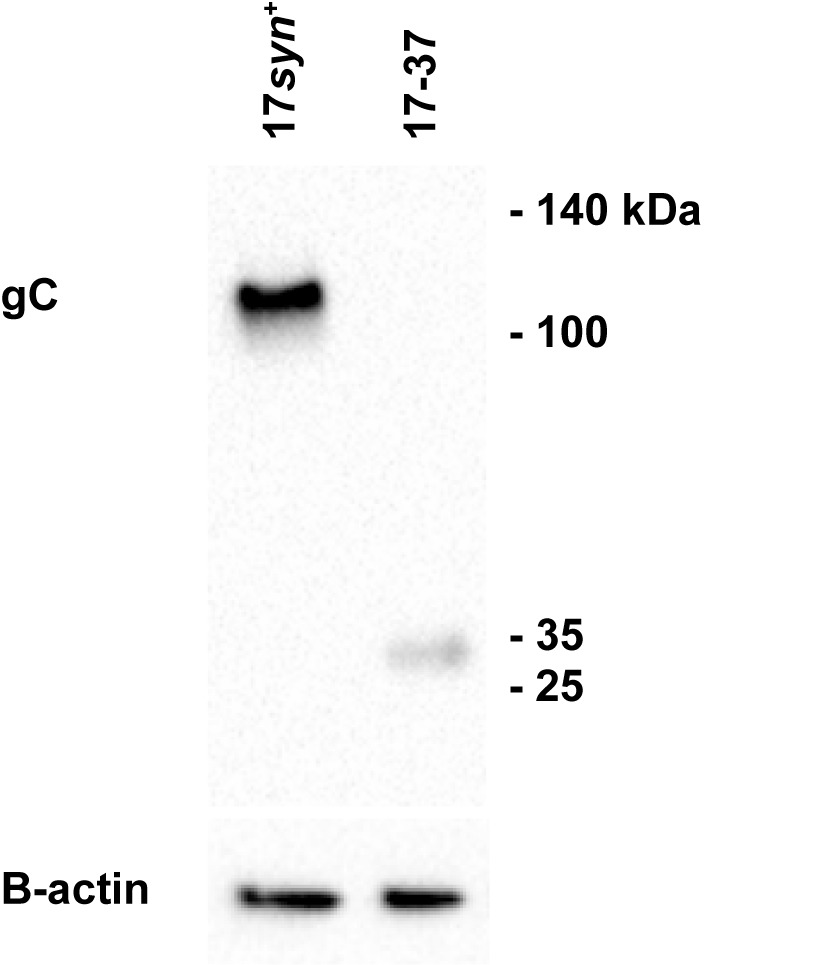

Supplement: S4 Fig — Western blot of gC in Vero cells infected with 17syn+ and 17-37 (MOI 1) at 24 HPI. 17-37 expresses a truncated form of gC. (TIF) [file ppat.1009166.s009.tif]
