# Supplementary material for: The latency-associated transcript locus of herpes simplex virus 1 is a virulence determinant in human skin
Source: PLoS Pathog. 2020 Dec 28;16(12):e1009166. doi: 10.1371/journal.ppat.1009166 (PMC7794027; doi:10.1371/journal.ppat.1009166)
Supplement: Attachment — Submitted filename: 2020-08-18 Response to Reviews.pdf [file ppat.1009166.s011.pdf]

August 18, 2020

Summary of Responses to Reviews:

- We have sequenced the genomes of the wild-type HSV-1 17*syn*$^+$ and seven derived viruses that we have used to assess the role of the LAT locus in skin virulence. We have added a comprehensive, comparative analyses of these datasets, accomplished through a collaboration that we formed with Dr Andrew Davison, MRC-University of Glasgow Centre for Virus Research, who we have added as a co-author.

- Based on the genome sequencing, we have found that a broad range of HSV-1 mutations have no impact on HSV-1 pathogenesis in human skin, and conversely, deletions in the 5' end of the LAT locus impair it.

- Our sequence analysis indicates that viral mutagenesis should always be accompanied with complete genome sequencing. Moreover, viruses that have been associated with contradicting results between different laboratories would be best to be sequenced.

- We have added experimental data evaluating two additional LAT locus mutants, which has confirmed our conclusion that the LAT locus is a determinant of HSV-1 virulence in human skin.

- We have added a skin explant model by which we show to that the skin pathogenesis defect in the xenograft model is not due to murine influences. This was accomplished through a collaboration with Dr Jennifer Moffat and Dongmei Liu, Department of Microbiology and Immunology, State University of New York-Upstate Medical University, who we have added as co-authors.

- We have performed laser capture microdissection of equal size skin lesions in combination with 3' end-specific RNA sequencing (3SEQ) to detect novel viral transcript candidates and to accurately compare viral transcript levels in skin infected with a wild-type or a LAT locus mutant virus. This was accomplished through a collaboration with Dr Joseph Foley, Department of Pathology, Stanford University, who we have added as a co-author. These data affirm that 17ΔN/H growth defect in human skin is not caused by differential transcript expression.

Reviewer's Responses to Questions

Part I - Summary
Please use this section to discuss strengths/weaknesses of study, novelty/significance, general execution and scholarship.

Reviewer #1: This paper reports the investigation of the phenotype of an HSV-1 LAT mutant (17 deltaN/H) in human skin xenografts implanted into SCID mice. Relative to WT HSV-1 strain 17 the LAT mutant is shown to have dramatically impaired infectivity. The authors conclude that the reported findings are paradigm shifting because LATs have been considered to be relevant only for HSV-1 latency and reactivation in neurons.
This is an observation of potential significance to the field but further confirmatory experiments with independent HSV-1 LAT mutants/revertants are required. 17 deltaN/H is unusual for a LAT deficient mutant in that it was originally reported to (a) express a novel 1.1kb transcript adjacent to the Not/Hpa deletion (b) to overproduce a 29Kda HSV protein and (c) produced small plaques in CV-1 cells (Block et al., (1993). It is unclear to me whether the dramatically reduced infectivity the authors report in human skin xenografts is due to the lack of LAT function, production of the 1.1kb novel RNA/29KDa product or the previously reported small plaques phenotype. These features of 17 deltaN/H are important, relevant to the current paper and should be considered. Also relevant is previous work demonstrating increased apoptosis of HSV-1 infected human immature dendritic cells in the absence of LAT using the 17 deltaN/H mutant and has therefore already implicated a phenotype of this mutant in cells other than neurons (Kather et al., (2010). J.Virol . 84:1034-1046). This paper should be cited and discussed.

Reply. Confirmatory experiments with other LAT mutants have been added as recommended.

We did not detect a 1.1 kb transcript but cannot definitely rule out its presence in epithelial cells since it could overlap with other transcripts. We have now quantified viral transcript expression by 3' end-specific RNA sequencing (3SEQ). 3SEQ was done through a collaboration with Dr Joseph Foley, Department of Pathology, Stanford University. These experiments indicate that transcriptional differences between the two viruses are not causative for the 17ΔN/H growth defect in skin, ruling out the immediate importance of such transcript or a protein in skin pathogenesis. We note, here, that Block *et al*. 1993 text describes that the 1.1 kb transcript is also present in 17*syn*$^+$ infection although at lower abundance and that the publication does not show uninfected controls.

We did not measure 17ΔN/H plaque sizes in either fibroblasts or primary keratinocytes in the absence of the rescuant virus that was used in the original Block *et al*. 1993 plaque size comparison in CV-1 cells. Recent publications describe that different 17*syn*$^+$ stocks show variability in plaque phenotype (Jones *et al*. 2019 J Gen Virology 100, doi:10.1099/jgv.0.001343) and that variability may increase during passaging (Kuny *et al*. 2020 Virus Evol. 6, doi:10.1093/ve/veaa013). Therefore, we sequenced the genomes of our 17*syn*$^+$ and 17ΔN/H virus stocks to correlate our skin pathogenesis findings with any sequence differences. We also showed that LAT locus mutations affect skin pathogenesis using two other mutant viruses.

We appreciate drawing our attention to the paper by Kather *et al*. (2010 J Virol., doi:10.1128/JVI.01409-09), which is now cited.

Other comments;
(1) Lines 110-116 (methods 413-420). There is no indication on the quality of the alignment, number of reads, number of replicates, parameters used in Seqman, the depth of coverage or any coverage graphs. Was there any evidence of low frequency SNPs?

Reply. As described in the overview of key changes to our manuscript, we have added a comprehensive, comparative analyses of eight viruses used in our study (Tables 1 and S1), in collaboration with Dr Andrew Davison. Detailed methods for our sequencing experiments have been added.

(2) The fact that there was incomplete coverage of some genes which were then confirmed to have no mutations by RNAseq raises questions on the quality from the genome sequencing.

Reply. Please see reply to (1).

(3) Did the authors detect the novel 1.1kb RNA previously reported to be transcribed by 17deltaN/H?

Reply. See reply to the summary above.

Reviewer #2: In this paper Vanni et al. report a new role for the complex and controversial Latency Associated Transcripts produced by Herpes simplex virus during infection. These transcripts, which are anti-sense to a number of key HSV genes such as ICP0 and ICP34.5, are the principle transcripts expressed during latent infection of neurons and have also been reported recently to have anti-apoptotic effects. AS indicated and partly confirmed by the authors, they have little effect on HSV replication in mouse skin and in cell lines as shown by a standard deletion mutant. Here the authors have compared infection of human skin xenografts, in a severe combined immunodeficiency mouse model, between a deletion mutant 17ΔN/H and the parental strain 17syn+, on replication and lesion formation. The mutant virus was carefully compared to wild type for potential functionally important adventitious mutations and none found. Examination of skin lesions was showed the presence of LAT transcripts in wild type but not in mutant virus infected skin. In the former the 2KB LAT intron was spliced as in latently infected neurons. Furthermore the LAT promoter regulated LAT transcription during productive infection. Ie LAT transcription was due to read through transcription from upstream viral promoters. Skin infected with wild type virus resulted in the usual lytic viral replication in epidermal cells, including those surrounding hair follicles, whereas the mutant virus had impaired infectivity and no evidence of cytopathicity.
The novelty of these observations lies in the authors claim that LAT functions are necessary for lytic replication of HSV in human (but not mouse) epidermis whereas in the past LATs were only considered relevant to HSV latency and activation in neurons.
In general the experiments are well done and support these important conclusions. However there are omissions and points of clarity (see below).
There should be a comment on why the skin xenograft is likely to be an accurate model of the effects of LATs in normal human skin free of murine influences. Appropriately they suggest that their results should be replicated in an ex vivo human epidermal explant system. However there are other examples of explants infected by HSV (eg foreskin explants), rather than needing to use a VZV reference (ref 52)

Reply. We appreciate the reviewer's assessment that our experiments supported our conclusions. We have now added skin explant culture data, through a collaboration with Dr Jennifer Moffat and Dongmei Liu, that confirm the 17ΔN/H growth defect in human skin free of any potential murine influences. We have also discussed the published data on LAT mutant viruses' phenotype in human foreskin fibroblasts (HFs) and added appropriate references. Our data on virus replication in uses HFs and primary keratinocytes agrees with this data. However, we do notice a defect in virus release for 17ΔN/H that is larger in keratinocytes than HF. We discuss how this may explain the previous findings.

Reviewer #3: The manuscript by Vanni et al from the Ann Arvin group convincingly communicate data to suggest the contribution of the HSV LAT promoter region to the pathogenesis of HSV-1 in human skin tissues harbored in the SCID-Hu Model. They show that at strain 17 virus lacking the known LAT

promoter regions is, in comparison to its strain 17 parent, attenuated for the growth and spread of virus in the human skin tissues. Virus grows particularly strong in the epithelial cell layers, as found in human lesions. They then show by RNA seq analyses that both viruses appear capable of expressing the majority of viral genes in human infected tissue, suggesting (but not proving) that the absence of LAT does not affect viral gene expression. Clearly the deletion of the LAT promoter regions does not affect growth in some human cell types (fibroblasts) but does in keratinocytes. As the authors suggest, this is a potentially paradigm shifting observation since up to this point (and largely based on mouse studies), we have largely assumed that LAT functions largely and predominantly as a neuronal specific regulator contributing to the HSV latent state and reactivation from it. I find these data intriguing and provocative, but have some important questions. Any mechanism is lacking.

Reply. We thank the reviewer's compliments. We hope to have addressed all the important points below.

Part II – Major Issues: Key Experiments Required for Acceptance
Please use this section to detail the key new experiments or modifications of existing experiments that should be absolutely required to validate study conclusions.

Generally, there should be no more than 3 such required experiments or major modifications for a "Major Revision" recommendation. If more than 3 experiments are necessary to validate the study conclusions, then you are encouraged to recommend "Reject".

Reviewer #1: 1. Verification of the observed phenotype with at least one independently derived LAT mutant/revertant.

Reply. We have verified the results using two other LAT promoter deletion viruses, 17ΔPst (which we found has a 1773 nt deletion in the 5' end of the LAT locus) and KOSΔPstLAT, coupled with 17ΔPstR and KOSΔPstR, respectively. We show that these two LAT mutants also have a growth defect in human skin (Figure 3). These results have reaffirmed our conclusion that the LAT locus is a virulence factor in skin.

2. As it stands there is insufficient description of the methods and reporting of the bio-informatics analyses to be able to verify the conclusions drawn in the manuscript.

Reply. We have modified the methods and rewritten the RNA sequencing result section as advised. We have added substantial new data of laser capture microdissection of equal size lesions in combination with 3SEQ to detect novel viral transcript candidates and to accurately compare transcript expression levels between 17*syn*$^+$- and 17ΔN/H-infected skin. We have explained this rationale in the reply to Reviewer #3 point 6 below. These data indicate that 17ΔN/H growth defect in human skin is not caused by differential transcript expression. We have also modified the text to describe the 17*syn*$^+$ and 17ΔN/H transcriptome results together to allow easier comparison between the two.

Reviewer #2: None

Reviewer #3: 1. This is potentially going to make a significant impact. As such, I feel there should be some data that guides the possible mechanism underlying the interesting observations. While it is clear there is a difference there is nothing indicating HOW LAT contributes to the differential growth of LAT- and + viruses in human tissue. The most obvious question that comes to mind is whether the purported attenuation of apoptosis by LAT is seen in human tissues infected with each virus. It would be relatively straightforward to stain for apoptosis markers or TUNEL on sections; apaptosis could easily affect spread and virus progeny yields.

Reply. We performed TUNEL on skin sections as recommended but found that all infected cells were positive for TUNEL signal, attributable to the fact that HSV-1 DNA is nicked during replication. Therefore, we've not included this data. We reference a publication from the Sawtell and Thompson labs, with TUNEL staining in neurones, showing that LAT increases neuronal survival during latency (2001 J Virol. 75, doi:10.1128/JVI.75.14.6660-6675). We have also added the reference Kather *et al*. (2010 J Virol., doi:10.1128/JVI.01409-09), as suggested by Reviewer #1, that has shown that 17ΔN/H virus has a defect in blocking apoptosis in primary dendritic cells.

With regard to the identification of a mechanism, we emphasise that whilst it has been shown that the LAT locus affects viral gene expression and inhibition of apoptosis in neurones, mechanisms by which LATs do this remain unknown, other than those of LAT-associated HSV-1 miRNAs. We did not identify comparable gene regulation functions for LAT in skin. In contrast to neurones, we show that the LAT locus dramatically affects viral replication and lesion formation in skin, with small effects on viral gene transcription.

We argue that our novel observations highlight the importance of renewed attention to the mechanisms by which the LAT locus contributes to either HSV-1 latency, lytic infection, or both. We predict that LATs will contribute to pathogenesis in both phases of HSV-1 infection through multiple mechanisms, some of which may be common to both differentiated cell types and others cell-type specific.

2. What happens to the mouse tissues? Did they do their virus to virus comparisons in the same mice strain that don't harbor the human skin? If the viruses are correct, they should not see any difference in peripheral growth. This is an important control that would support their conclusion of a human tissues specific role. Do the mice become infected with each virus upon say a skin infection, and develop a comparable lesion, aand are there signs of spread to the mouse and replication in the tissue capsules harboring and adjacent to the human skin implant?

Reply. This was a very pertinent question that caused us to realise our description of the xenograft model was deficient. The human skin xenografts are implanted subcutaneously so that no mouse skin cells are in contact with the human skin. A fibrous membrane forms around the graft but the human tissue block is dissected away from this capsule when the xenograft is retrieved and processed to prepare sections like those shown in Figure 2. This membrane can get infected with the virus. After being amplified in the skin xenograft, the virus does spread to the mouse CNS through blood. In SCID mice with limited immune responses, mortality is seen shortly after ~14 days post-infection with 17*syn*$^{+}$. In contrast, such was not observed for 17ΔN/H even after high inocula infections presumably due to the growth defect in skin. We have referred to the spread to the mouse in lines 254-257.

The scope of our study is to investigate HSV-1 pathogenesis in human skin epithelium, using primary cells and tissues from the host. We used human skin xenografts *in vivo*, cultured skin explants, and primary skin keratinocytes, and asked what would happen if LATs were not present. While some LAT locus mutant viruses might have a defective phenotype in mouse skin too, our conclusion about the importance of the LAT locus for replication and lesion formation in human skin would not be affected. We address this point in the Discussion, speculating that these mutants might replicate normally given findings with a LAT deficient virus expressing luciferase reporter (Nicoll *et al.* 2016 PLoS pathogens 12, e1005539) and we also describe why LATs may have species-specific functions in human skin.

3. This then brings up the possible differences in innate responses to the virus infection. It is feasible that LAT is contributing to the innate resistance environment, the responses to (and possible induction of) IFNs and the intact innate immune components that remains in the SCID-hu mice. Are tissue infiltrated by Macrophages and neurotrphils (and NK cells if they are intact)? Some of this might come

from the analyses of murine host changes associated with the implants, which does not seem to have been analyzed or considered for analyses.

Reply. As explained above, we have confirmed the defect in 17ΔN/H growth also in skin explants free of any murine responses to address this concern. It is possible that NK cells and monocytes might infiltrate skin xenografts in SCID mice although this has been observed at time points later than those we used. The level of infiltration would be expected to be the same in 17*syn*$^{+}$ and 17ΔN/H infected xenografts.

4. While this is decidedly niggly, I have to state that almost all strong published HSV -1 studies compare a viral mutant to the restored virus (in which the mutation is corrected in order to discount the contribution of spurious mutations top the phenotype). While sequencing was done on both viruses and there was no reported virus differences, there may be mutations (altered repeats, packaging elements and ICP34.5 for example, which can often be in very-difficult-to-sequence regions) that were not identified in the sequencing analyses. I wish this type of comparison had been done, and while they have taken steps to address spurious mutation contribution, The work will still be faulted by many investigators in the HSV field for not being done here.

Reply. Our decision to undertake comprehensive genome sequencing was in response to this important point. The investigator who made the 17ΔN/H rescuant no longer had the virus. We have modified the text to clearly state that the rescuant is not available. Therefore, we have sequenced our stock of the wild-type 17*syn*$^{+}$ and 17ΔN/H and six additional 17*syn*$^{+}$-derived viruses and have completed an extensive comparative genome analysis in collaboration with Dr Andrew Davison. This work is now presented in the detail in the first Results section and in Tables 1 and S1.

While the genomic approach became necessary for our study because the 17ΔN/H rescuant could not be obtained, there are significant implications beyond our investigation of the LAT locus. Critically, we found that generation of mutant and rescuant pairs of viruses, by whatever means, should always be accompanied with genome sequencing along with the parental virus. We have incorporated this major point into the Discussion.

In addition, we have verified the results using two other LAT promoter deletion viruses.

5. The promoter deleted viruses appear to show a growth/no growth phenotype. Could it be that this indicates an unexpected bottleneck to the infection process? Worth discussion.

Reply. Yes, we do believe there to be a bottleneck in the replication as a few xenografts infected with 17ΔN/H do have detectable viral titres. As suggested, we have now discussed this in lines 220-223, 250-251, and 586-587.

6. I note that from the mapping studies there are several notable nearby transcriptional differences. Indeed UL56 seems proportionally very low. There appear to be additional transcripts mapping just upstream of UL56 that are much lower. Of note is the reports of a RNA (the initial AL RNA) and possible small open reading frame by Perng et al 2002 that reads antisense to LAT that has been reported to encode a protein that contribute to murine and rabbit pathogenesis on HSV.

Reply. In response to the comment on UL56 transcript expression, we have clarified the text to explain that our RNA-seq experiment was designed to investigate whether all known HSV-1 transcripts are expressed in skin. Importantly, differences in lesion formation during skin pathogenesis of wild-type and mutant viruses presents a multifaceted challenge in quantifying and comparing the transcriptomes in a biologically relevant manner. Smaller lesions in the tissues infected with 17ΔN/H (Fig. 2) result in lower amounts of viral RNA (Fig. 5). For this, a simple normalisation to total transcripts is not a

sufficient approach, as the number of newly infected and late infected or lysed cells depends on lesion size. This in turn may affect the proportion of transcripts from different kinetic classes (immediate-early, early, and late) present. We have added this explanation into the text.

We have addressed this problem by quantifying viral transcripts using laser capture microdissection on equal-size skin lesions in combination with 3' end-specific RNA sequencing (3SEQ) in order to best represent the same stage of infection for more accurate quantification of the transcriptomes (Tables S4 and S5). We did find 3 transcripts to be significantly different between 17*syn*$^+$ or 17ΔN/H. However, these transcripts were not differentially regulated between 17ΔN/H and two other viruses that are not defective in skin, supporting the conclusion that the transcriptional differences were not responsible for the 17ΔN/H growth defect.

We did not observe transcription indicative of the presence of AL-RNA (lines 382-383 and 404-405). However, 0.7 kb LAT is expressed during skin infection and the implications of this transcript and its potential protein's presence are discussed.

Part III – Minor Issues: Editorial and Data Presentation Modifications
Please use this section for editorial suggestions as well as relatively minor modifications of existing data that would enhance clarity.

Reviewer #1: The RNA Seq methods (lines 432-452) need to be carefully proof read and correctly referenced.

Reply. We've detailed the RNA-seq methods.

Lines 408-409 should be re-phrased to clarify Paul Khavari's role in the provision of foreskin keratinocytes!

Reply. We've rephrased this section.

Reviewer #2: Results
line 114: This sentence is unclear.

Reply. We've added new genome sequencing data and discussed all differences between 17ΔN/H and 17*syn*$^+$ in detail.

Figs 2D/E Lines 146- 160 is confusing: in line 141 HSV DNA from mutant virus DNA is said to be present at 11dpi but in line 157 it is at 14dpi (?in hair follicles). I can only see it at 14 dpi. Arrows would help and also the absence of artefact from hair staining. The superficial epidermis is not shown in this figure either. In Fig 2D wt 11 dpi there appears to be LATS staining in hair follicles without DNA staining. How can this be explained?

Reply. We agree with the reviewer. In general, correlating the architecture across sections is difficult because of the three-dimensional nature of the tissue, e.g. not all hair follicles are oriented on the same plane. To clarify the information presented in Figure 2, we have added a diagram of the different tissue layers and features and inserted asterisks across the different panels to indicate the corresponding locations as well as symbols to indicate sectioning artefacts, lysed epithelial cells, and cytopathic effect (+, [], and #). The skin epidermis is not present in the 17*syn*$^+$-infected skin as the virus lyses all epithelial cells generally by 7 DPI. This is in contrast to 17ΔN/H virus whose pathogenesis is defective due to the deletion at the 5' LAT locus. We hope that the information is more accessible with these revisions.

Fig 2 F/G There is little explanation why they have the compared PML staining between mock, wt and mutant. The reasons for showing these results and their significance need to be further explained

Reply. We've changed the text to better explain the importance of the PML staining.

Line 251 to 253: why are the p values expressed as intervals rather than exact numbers?

Reply. We've added the exact p values.

In the RNAseq experiments were there any differences in the relative expression of late and early HSV transcripts between wild type and mutant virus?

Reply. The reviewer makes a fair assessment of the importance of the relative expression of late and early HSV transcripts between wild type and mutant virus. The number of newly infected and late infected or lysed cells depends on lesion size. This in turn may affect the proportion of transcripts from different kinetic classes. To address this, we performed laser capture microdissection in combination with 3SEQ on equal-size skin lesions infected with the viruses, as described above in the reply to Reviewer #3 point 6.

Discussion
line 372: The differential effects of ICP47 on cellular MHCI expression in mice and humans is also an excellent example of species specificity.

Reply. We've added the reference (Ahn *et al*. 1996 EMBO journal 15, 3247-3255).

Are there any clues, perhaps from human RNA seq results about why LAT has different effects on viral replication in mouse and human skin? Is the congruence in plaque size but difference in viral release between wild type and mutants virus indicative of differential effects on cell to cell vs cell free spread?

Reply. We are not aware of a comparison of human and mouse skin cell transcription profiles that might provide such clues. However, we have discussed important differences in skin architecture that may be related. We agree that differences in cell-cell spread cell free spread could play a role even though viral gene transcription did not differ, especially because the xenograft environment is more restrictive and representative of the clinical infection where cell-to-cell spread is important. We have added additional text to highlight the virus release data. However, we are cautious about drawing direct conclusions about this being the cause for the growth defect as the release phenotype in keratinocytes is significant but much smaller in magnitude compared to the 8-log effect of the LAT mutant on skin growth. We have noted that this may reduce skin growth but would likely account only in part for the severe growth defect.

Reviewer #3: For clarity and ease of communication, please add to figure 3A/B a diagram showing the genome and position of the terminal and internal repeat regions and the LAT loci in the genome.

Reply. We have added annotations for the repeat regions in Figures 1 and 5 (formerly Fig. 3). In Figure 1, we have also annotated the mutations/deletions in the different viruses. In Figure 5B and D, we have now clearly marked the area deleted in the 17ΔN/H virus. We have also fully aligned the sequences in Figure 5C and D, show the genomic positions, and have annotated three 3SEQ region hits that aligned in the proximity of the LAT locus (3SEQ9, 3SEQ10, and 3SEQ11 marked with *, †, and ‡). Two of these, 3SEQ10 and 3SEQ11 are deleted in the 17ΔN/H. Further, we have clarified the Figure 5 and Tables S4 and S5 legends to explain which areas we are comparing.

8. Would I be correct in thinking I see a rather large difference in the transcripts of the very terminal

regions between the two viruses at both ends?

Reply. We address this point in our reply to Part II Reviewer #3 point 6.

9. Please shorten the discussion, it has a lot of speculation and since there is no real underlying mechanism revealed in this work, it tends to come over as an attempt for a “catch all cases”.

Reply. We agree and have made major revisions to remove repetition and highlight key points more concisely.

10. Line 166. Stating that ICP0 expression is not unaffected by the deletion, based on only IF data, is overconcluding. There could easily be significant differences in infected cells in protein levels that are not seen with IF

Reply. We have clarified that we are not trying to quantify ICP0 expression in Figure 2. We have used the immediate-early protein ICP0 as a marker of early infection, as lesions in 17ΔN/H-infected tissue are extremely difficult to find even at 11-14 days due to the growth defect. We do note that the amount of polyadenylated ICP0 transcript during the 17ΔN/H infection was not changed (Figure 5), which indicates that differences in ICP0 protein level are not likely.

We thank all three reviewers for their helpful comments and hope that the data we have added respond to their valuable critique.
