# Supplementary material for: The latency-associated transcript locus of herpes simplex virus 1 is a virulence determinant in human skin
Source: PLoS Pathog. 2020 Dec 28;16(12):e1009166. doi: 10.1371/journal.ppat.1009166 (PMC7794027; doi:10.1371/journal.ppat.1009166)
Supplement: Attachment — Submitted filename: 2020-11-03 PPATHOGENSD2001788 response to the reviewers.docx [file ppat.1009166.s012.docx]

Reviewer Comments (if any, and for reference):

Reviewer's Responses to Questions

**Part I - Summary**
Please use this section to discuss strengths/weaknesses of study, novelty/significance, general execution and scholarship.

Reviewer #1: Figures 3D-G are numbered correctly in figures and legends but incorrectly in the body of the Results.

This point was raised by all three reviewers. Figure 3 was missing one panel on viral titres recovered from xenografts infected with high 17∆N/H virus inocula. We’ve added the panel as Fig. 3C. We’ve modified the legend and text accordingly.

Please clarify that the significant results at 4dpi which become insignificant at 7 (?not 8) dpi are due to lack of residual keratinocytes destroyed by cytopathic spread of wt virus?

We’ve corrected the text to say 8 dpi like in the figure.

We’ve clarified the text describing the relationship between viral titres and cytopathic spread in skin. It now says: “In the higher inoculum experiments and those with longer time intervals post-infection, 17*syn*^+^ titres were lower in both xenograft and *ex vivo* models due to epithelial cell depletion, which left no epithelial cells to support viral replication (**Fig. 2-3** and data not shown).”. This is now located after the ex vivo results paragraph as it relates to both models.

Figure 3E adds little as the results are not significant for the reasons explained. Either further replicates are needed for even nonparametric statistics or the results deleted or presented as supplementary figures. The two other LATS mutants are sufficiently convincing.

We’ve moved the KOS∆PstLAT data to the supplementary as the new Figure S3 as a response to this suggestion. We’ve modified the Figure 3 numbering in the text accordingly.

Reviewer #2: The revised manuscript, titled “The Latency-Associated Transcript Locus of Herpes Simplex Virus 1 is a Virulence Determinant in Human Skin”, reports that HSV-1 viruses mutated to interfere with viral transcription in the LAT locus can’t spread in human skin xenografts and cultured skin explants. The observation is interesting and potentially significant in the HSV-1 pathogenesis. The careful analysis and comprehensive comparison on wide-type and mutant viral genomes is to be praised in the detailed characterization of virus phenotypes.

Cheers very much for these positive comments!

Prior to this study, LAT has not been implied in the lytic infection of HSV life cycle. The authors showed that deletions of the upstream of LAT promoter and 5’ LAT region, as depicted in the strain 17ΔN/H, severely impaired HSV replication and lesion formation in skin. However, this defect in virus spread was caused neither by the defect in 2kb LAT production, which is the hall mark of HSV latency, nor by many nonsynonymous mutations found in the viral coding regions. Based on detailed mapping on the transcripts produced in the LAT locus, it seems like the 0.7 kb transcript upstream of LAT promoter and/or the 5’ exon sequences in the mLAT could be responsible for the observed phenomenon. The author added KOSΔPST mutant and confirmed the defective phenotype. But the mutation in ΔPST overlaps with 0.7kb transcript, so as 17ΔN/H. Since the 0.7kb transcript has been suggested in the virulence of HSV-1 17syn+, further evaluation using mutants separating in the two regions might be needed in providing clarity on the role of LAT in HSV-1 skin pathogenesis.

We agree. This is a very interesting question.

Reviewer #3: The authors have made significant improvements to this paper, which now provides compelling evidence that the HSV-1 latency associated transcripts act as a virulence determinant in human skin. The inclusion of additional LAT mutant viruses and, where available, revertant viruses strengthens their message. The inclusion of full sequence data for the mutant viruses will also act as a wake-up call to research groups working in this complex field. This is an important body of work that adds important new information concerning the role of LATs in pathogenesis outside of known functions in neuronal cells.
The authors should carefully review Figure 3, and the text describing this Figure in the results - it appears that some panels are miss-labelled/missing.

We really appreciate these comments! We strongly believe that all laboratory virus strains, whether they are made by homologous recombination or using BACs, should be sequenced.

**Part II – Major Issues: Key Experiments Required for Acceptance**
Please use this section to detail the key new experiments or modifications of existing experiments that should be absolutely required to validate study conclusions.

Generally, there should be no more than 3 such required experiments or major modifications for a "Major Revision" recommendation. If more than 3 experiments are necessary to validate the study conclusions, then you are encouraged to recommend "Reject".

Reviewer #1: (No Response)

Reviewer #2: (No Response)

Reviewer #3: (No Response)

**Part III – Minor Issues: Editorial and Data Presentation Modifications**
Please use this section for editorial suggestions as well as relatively minor modifications of existing data that would enhance clarity.

Reviewer #1: (No Response)

Reviewer #2: There is mixed use of LATs with LAT locus in the Author Summary, starting on line 44. Since the LAT splicing mutant have no effect in skin virulence, plus it is unknown whether other transcripts in the LAT promoter region express in the latency, it is more precise to use LAT locus than LATs, to avoid misleading.

As the reviewer pointed out, using “LATs” at points in the Author summary was inaccurate. We’ve changed the text to say LAT locus rather than “LATs” since the LAT splicing mutant had no effect in skin virulence.

Fig. 3C, Fig. 3D, 3E, 3F, 3G, seem shifted, the text and figure don’t match. There is one panel missing in Fig. 3.

As said above as a response to reviewer #1, we’ve added the missing panel into Figure 3. We’ve also moved the KOS titre data to the supplementary and made changes to the figure legends and text accordingly.

Line 357-359 needs modification. Fig. 1 and 2C didn’t verify ICP0 retained its ubiquitin ligase function. Fig.2C only showed ICP0 expression.

We’ve removed lines 357-359 describing Fig. 2C which said that we see degradation of PML by ICP0. We now refer to PML as a cellular marker.

Line 411-412 needs modification. Three references (47, 49, 50) only used 17 and KOS strain in cell culture. No mention on F strain.

We’ve removed the F strain mention from the text.

Reviewer #3: The authors should carefully review Figure 3, and the text describing this Figure in the results - it appears that some panels are miss-labelled/missing.

We’ve addressed this comment as described above.

Summary of responses to reviews:

- Changed the Author Summary text starting at line 44 to say LAT locus rather than “LATs” since the LAT splicing mutant had no effect in skin virulence.
- Added missing 'NH high inocula' panel to Fig. 3 that all reviewers had spotted. This is a high inocula deltaN/H infection experiment - now Figure 3C
- Moved Figure 3E (KOS skin titres) to supplementary figures
- Updated Figure 3, legend, and text describing these results accordingly
- As KOS titre figure is now in the supplement as Figure S3, the gC figure location changed to S4. The text has been updated accordingly
- Corrected the ex vivo skin titre "7 DPI” to “8 DPI” in the text to match the ex vivo skin titre figure (Figure 3D)
- Clarified the text to say that the significant results in skin titres at early time points become insignificant later in the time-course due to lack of residual keratinocytes destroyed by cytopathic spread of wt virus. The sentence has been moved to a more appropriate place in the text
- Removed lines 357-359 describing Fig. 2C which said that we see degradation of PML by ICP0 & describe PML as a cellular marker
- Removed F strain mention from line 411-412 as the references (47, 49, 50) only used 17 and KOS strain

Additional changes:

- Table 1 & S1 were merged as Table 1 too large to fit into the main text. Table 1 amino acid changes are now the first spreadsheet of the Table S1. The other spreadsheets remain as they were. Table S1 legend and references to it in the text have been modified
- Added grant information e.g. website links to funding section
- Corrected typos
- Adjusted SEM bars in Figure 4 & corrected the exact p values in the legend
- Table S2 Salmon numbers corrected as the total TPT of wild-type did not equal 1,000
- Added “The numerical data used in all figures are included in Data S1 except for the genome sequencing, which can be found in Table S1.” as the last line of the methods section
- Made Data S1 table with raw values for virus titre and RNA-seq experiments. The virus titre information additionally includes n number, average, and SEM values. Genome sequencing raw values are found in Table S1. Genome and RNA-seq datasets can be downloaded from the NCBI databases referenced in the text
- Pasted Table 1 into the main text
- Removed TM from NetWell^TM^
- Removed “(data not shown)” from “A low inoculum (1,000 PFU) caused lesions in all 17syn+-infected xenografts and none of the 17∆N/H-infected xenografts at 7 DPI (data not shown).” as representative panels are included in Figure 2B & D and Figure 2D. Fig. 2 legend now says “representative”.

PLOS authors have the option to publish the peer review history of their article ([what does this mean?](https://journals.plos.org/plospathogens/s/editorial-and-peer-review-process#loc-peer-review-history)). If published, this will include your full peer review and any attached files.


If you choose “no”, your identity will remain anonymous but your review may still be made public.


**Do you want your identity to be public for this peer review?** For information about this choice, including consent withdrawal, please see our [Privacy Policy](https://www.plos.org/privacy-policy).

Reviewer #1: No

Reviewer #2: No

Reviewer #3: No

Figure Files:

While revising your submission, please upload your figure files to the Preflight Analysis and Conversion Engine (PACE) digital diagnostic tool, [https://pacev2.apexcovantage.com](https://pacev2.apexcovantage.com/). PACE helps ensure that figures meet PLOS requirements. To use PACE, you must first register as a user. Then, login and navigate to the UPLOAD tab, where you will find detailed instructions on how to use the tool. If you encounter any issues or have any questions when using PACE, please email us at [figures@plos.org](mailto:figures@plos.org).

Data Requirements:

Please note that, as a condition of publication, PLOS' data policy requires that you make available all data used to draw the conclusions outlined in your manuscript. Data must be deposited in an appropriate repository, included within the body of the manuscript, or uploaded as supporting information. This includes all numerical values that were used to generate graphs, histograms etc.. For an example see here: <http://www.plosbiology.org/article/info%3Adoi%2F10.1371%2Fjournal.pbio.1001908#s5>.

Reproducibility:

To enhance the reproducibility of your results, PLOS recommends that you deposit laboratory protocols in [protocols.io](http://protocols.io), where a protocol can be assigned its own identifier (DOI) such that it can be cited independently in the future. For instructions see [http://journals.plos.org/plospathogens/s/submission-guidelines#loc-materials-and-methods](http://journals.plos.org/plospathogens/s/submission-guidelines)

*In compliance with data protection regulations, you may request that we remove your personal registration details at any time.* [*(Remove my information/details)*](https://www.editorialmanager.com/ppathogens/login.asp?a=r)*. Please contact the publication office if you have any questions.*
